# *Ganoderma microsporum* immunomodulatory protein, GMI, promotes C2C12 myoblast differentiation *in vitro* via upregulation of Tid1 and STAT3 acetylation

**DOI:** 10.1371/journal.pone.0244791

**Published:** 2020-12-31

**Authors:** Wan-Huai Teo, Jeng-Fan Lo, Yu-Ning Fan, Chih-Yang Huang, Tung-Fu Huang

**Affiliations:** 1 Institute of Oral Biology, National Yang-Ming University, Taipei, Taiwan; 2 Department of Dentistry, School of Dentistry, National Yang-Ming University, Taipei, Taiwan; 3 Cancer Progression Research Center, National Yang-Ming University, Taipei, Taiwan; 4 Department of Dentistry, Taipei Veterans General Hospital, Taipei, Taiwan; 5 Department and Institute of Pharmacology, School of Medicine, National Yang-Ming University, Taipei, Taiwan; 6 Graduate Institute of Chinese Medical Science and Institute of Medical Science, China Medical University, Taichung, Taiwan; 7 Institute of Basic Medical Science, China Medical University, Taichung, Taiwan; 8 Department of Health and Nutrition Biotechnology, Asia University, Taichung, Taiwan; 9 School of Medicine, National Yang-Ming University, Taipei, Taiwan; 10 Department of Orthopedics and Traumatology, Taipei Veterans General Hospital, Taipei, Taiwan; New York Medical College, UNITED STATES

## Abstract

Ageing and chronic diseases lead to muscle loss and impair the regeneration of skeletal muscle. Thus, it’s crucial to seek for effective intervention to improve the muscle regeneration. Tid1, a mitochondrial co-chaperone, is important to maintain mitochondrial membrane potential and ATP synthesis. Previously, we demonstrated that mice with skeletal muscular specific Tid1 deficiency displayed muscular dystrophy and postnatal lethality. Tid1 can interact with STAT3 protein, which also plays an important role during myogenesis. In this study, we used GMI, immunomodulatory protein of *Ganoderma microsporum*, as an inducer in C2C12 myoblast differentiation. We observed that GMI pretreatment promoted the myogenic differentiation of C2C12 myoblasts. We also showed that the upregulation of mitochondria protein Tid1 with the GMI pre-treatment promoted myogenic differentiation ability of C2C12 cells. Strikingly, we observed the concomitant elevation of STAT3 acetylation (Ac-STAT3) during C2C12 myogenesis. Our study suggests that GMI promotes the myogenic differentiation through the activation of Tid1 and Ac-STAT3.

## 1. Introduction

Skeletal muscle, wrapped with the connective tissues, constitutes of myotubes that bundles into myofibrils. It comprises about 40% to 45% of our body weight and enables our body to maintain the posture and to perform a wide range of movements, motions and stability [1,2]. Skeletal muscle possesses regenerative capability against minor injury, where the satellite cells differentiate into myoblasts followed by fusion of myoblast into multinucleated myotubes to replace the injury myofibers [1,3,4]. However, ageing or chronic diseases such as atrophy, cachexia and sarcopenia will lead to muscular loss and the deficiency to maintain or restore the normal structure and function of the impaired muscle [2,5,6]. Hence, muscle regeneration and transplantation of myogenic cells is an essential therapy for the muscular dystrophies.

Skeletal muscle differentiation is a multi-stage highly-regulated process that includes myoblast division, elongation, and fusion [7]. Along with myogenesis process the expression of stem cell markers such as Pax7 is gradually lost. Meanwhile, appearance of differentiation markers, such as myogenin, myosin heavy chain (MyHC) and muscle-regulatory factor 4 (Mrf4) are gradually increase [8]. Apart from this, previous studies demonstrated that interleukin 6 (IL-6) a pro-inflammatory cytokine, is elevated in response to muscle contraction [9]. IL-6 is found to participate in muscle regeneration in muscular dystrophy [10]. Additionally, it has been reported that IL-6 promote myogenic differentiation of C2C12 via signal transducer and activator of transcription 3 (STAT3) axis. Knockdown of IL-6 and STAT3 in C2C12 differentiating cells suppress the expression of the myogenic markers myogenin and MyHC, consequently, resulting the inhibition of myotube fusion. Furthermore, primary muscle cells isolated from IL-6 knockout mice feature a significant reduction of MyHCIIb positive cells [11].

Tumorous imaginal disc 1 (Tid1) is a mammalian mitochondrial DNAJ/HSP40 co-chaperone protein homolog to the Drosophila tumor suppressor protein Tid56 [12]. Tid1 protein contains a conserved J-domain by which to interact with heat shock protein 70 (HSP70) family members via stimulation of ATPase activity [13]. *Tid1* transcripts are expressed in two alternatively splicing isoforms, Tid1-long form (Tid1-L) and Tid1-short form (Tid1-S). Interestingly, Lu *et al*. reveal that Tid1-L exhibit higher cytosolic stability and a slower rate of mitochondrial import in comparison with Tid1-S. Further, they report that Tid1 interacts with the HSP70 substrate proteins, STAT1 and STAT3 [14].

Tid1 has been identified as a tumor suppressor [15–19]. Previously, we have demonstrated that cell proliferation is inhibited in Tid1 overexpressed head and neck squamous cell carcinoma (HNSCC) cells. Conversely, *Tid1* gene knockdown enhances cancer cell malignancy such as cell migration and invasion *in vitro*, and tumorigenicity *in vivo* [15]. Additionally, Tid1 abrogates the Galectin-7/TCF3/MMP9 axis to repress the cancer metastasis [16]. We also report the participation of Tid1 in early embryogenesis [20], T cells development [21], muscular development and mitochondrial biogenesis [22] in normal development. We observe the elevation of Tid1 protein in differentiated C2C12 myoblast. In opposite, *Tid1* gene knockdown impairs the differentiation ability of C2C12 myoblasts. Concurrently, we perceive the reduction of intracellular ATP amounts and mitochondrial activity which results in energy imbalance and promotion of cells apoptosis. Moreover, our established HSA-Tid1^*f/f*^ and HSA-Tid1^*f/+*^ transgenic mice (mice with Tid1 deficiency specifically in skeletal muscle) show severe muscular dystrophy with reduced motor activity, accompanied with impairment of activity of ATP sensor (p-AMPK) and mitochondrial biogenesis protein, peroxisome proliferator activated receptor gamma coactivator-1 alpha (PGC-1α) [22]. Thus, activation of Tid1 is important to maintain the integrity of mitochondrial and myogenesis of skeletal muscle.

*Ganoderma* is a genus of polypore fungi widely used as medicinal purposes for centuries, particularly in China, Japan and Korea [23], and commonly called as Lingzhi. In accordance with the theory of traditional Chinese medicine, *Ganoderma* possesses the ability to strengthen body resistance and consolidate the constitution [24]. GMI, an immunomodulatory protein cloned from *G*. *microsporum*, is found to exhibit anti-inflammatory effect. GMI has also been studied in a broad-spectrum application for the anti-cancerous treatment through the regulation of immune system. GMI inhibits tumor necrosis factor alpha (TNFα)-mediated matrix metallopeptidase 9 (MMP-9) expression and migration in A549 cancer cells [25]. Furthermore, Hsin *et al*. shows that GMI has the ability to inhibit tumorigenicity and induce autophagy cell death in non‐small lung cancer cell lines [26–29]. The anti-cancerous roles of GMI have also been studied in urothelial carcinoma cells [30], oral carcinomas stem cells (OCSCs) [31] and human fibrotic buccal mucosal fibroblasts (fBMFs) [32]. Despite these, the biological function of GMI is seldom been discussed in normal circumstances.

Inflammatory responses are critical in skeletal muscle myogenesis process. Previous study has reported that the transition of M1 to M2 macrophage releases the anti-inflammatory cytokines to promote skeletal muscle differentiation [33]. Moreover, for IL-10 knockout mice, with the deficiency of the well-known anti-inflammatory cytokine, display muscle loss and develops muscle weakness [34]. However, this frail model is reversed by up taking grape seed extract [35], which is known for its anti-oxidative and anti-inflammatory effect [36,37].

Since GMI is found to possess the anti-inflammatory effect; therefore, in this study, we explored the promotion of GMI in skeletal muscle myogenesis. Indeed, treatment of GMI in C2C12 myoblast promoted the differentiation of myoblast and fusion into myotubes. Additionally, we observed the elevation of Tid1, PGC-1α, and Acetylated-STAT3 (Ac-STAT3) proteins in the differentiated myoblast. Our findings provide a new insight of GMI treatment to promote C2C12 myoblast differentiation via activation of Tid1 mitochondria co-chaperon and Ac-STAT3.

## 2. Materials and methods

### 2.1 Myoblast cell line and myogenesis induction

Mouse C2C12 myoblasts (BCRC, 60083) were obtained from Bioresourse Collection and Research Center (Hsinchu, Taiwan). The cells were expanded in growth medium of Dulbecco’s Modified Eagle Medium (DMEM), 10% fetal bovine serum (FBS), 1% L-glutamine and 1% Penicillin-Streptomycin Amphotericin (PSA) at 37°C under 5% CO_2_. When cells reached 90% -100% confluence, the growth medium was replaced with differentiation medium consisting of DMEM, 2% horse serum (#16050–130, Thermo Fisher Scientific, New Zealand), and 1% PSA. Cell culture media and supplements were purchased from biological industries (BI, Israel).

### 2.2 Generation of the skeletal muscle specific Tid1 deletion mice

Tid1 floxed mice were generated according to previous study [20]. Mice with Tid1 gene homozygous or heterozygous deletion specifically in skeletal muscle were generated by crossing *Tid1*^*f/f*^ or *Tid*^*f/+*^ mice with transgenic HSA-Cre mice. The genotyping of the HSA-Cre transgene and the Tid1-deficient mice (*HSA-Tid*^*f/f*^ or *HSA-Tid*^*f/+*^) was performed by polymerase chain reaction (PCR) using genomic DNA isolated from the tail.

### 2.3 GMI protein

GMI protein was manufactured by MycoMagic Biotechnology Company Ltd (Taipei, Taiwan). During experimental conduction, the GMI sample was dissolved in differentiation medium to reach different concentrations.

### 2.4 Isolation and culture of murine primary myoblast

Primary myoblasts were isolated as described as previous study [38]. Skeletal muscle tissues were minced and digested with digestion medium (high glucose DMEM, 1% PSA, 2.5% HEPES, and 400 U/ml collagenase II). The digested tissues were centrifuged, and the muscle pellet was subsequently resuspended with neutralizing/isolation medium (NIM, high glucose DMEM, 10% FBS, and 1% PSA). The muscle pellet mixtures were filtered twice, firstly through the 70 μm strainer and followed by 30 μm strainer to get the cell mixture. The cell mixture was centrifuged and resuspended in myoblast growth medium (MGM, F-10 media, 20% FBS, 1% PSA, and 10 ng/ml fibroblast growth factor). After 72 hours cultured on a 10% matrigel-coated dish, the cells were trypsinized and passaged until the cells >95% myoblast purity was achieved.

### 2.5 Cell cytotoxicity assay

C2C12 myoblasts were counted and approximately 5.5 x 10^4^ cells per well were seeded in a 24-well plate. The cells were expanded in growth medium of Dulbecco’s Modified Eagle Medium (DMEM), 10% fetal bovine serum (FBS), 1% L-glutamine and 1% Penicillin-Streptomycin Amphotericin (PSA) at 37°C under 5% CO^2^. When cells reached 90% -100% confluence, the growth medium was replaced with differentiation medium consisting a series concentrations of GMI (0, 0.01, 0.05, 0.1, 0.5, 1, 5, 10, 20 and 30 μg/ml). Three replicates were made for each measurement. The cells we pre-treated with GMI for 24 hours. 1/10 of the CCK-8 reagent (Dojindo, Japan) was added into each well, and O.D at 450 nm was measured using Spark® Multimode Microplate Reader (Tecan Trading AG, Switzerland) after 2 hours incubation at 37°C.

### 2.6 Immunoblot analysis

The cells crude proteins were extracted with RIPA buffer. The protein concentration was quantified through the Protein Assay Dye Reagent (Bio-Rad, USA). The extracted proteins were loaded onto SDS-polyacrylamide gels for electrophoresis and then transferred to nitrocellulose membranes. The nitrocellulose membranes were blocked with TBST containing 5% skimmed milk for 1 hour at room temperature followed by incubation with the corresponding primary antibodies and secondary antibodies. The signals were visualized by the enhanced chemiluminescence system as described by the manufacturer (Millipore, Germany) in conjunction with in LAS-4000 image analyzer (GE Healthcare, Japan). Mouse anti-MyoD (MA1-41017) and rabbit anti-Acetyl-STAT3 (Lys685) (PA5-17429) were purchased from Thermo Fisher Scientific (USA). Mouse anti-Tid1 (RS13) (sc18819) and rabbit anti-IL6 (M-19) (sc-1265) were purchased from Santa Cruz (USA).

Mouse anti-MyHC (05–716), mouse anti-PGC-1α (ST1202) were from Sigma-Aldrich, USA, rabbit anti-p-STAT3 (Tyr705) (Cell Signaling, USA). Rabbit anti-total STAT3 [C3] (GTX104616) and rabbit anti-beta actin (GTX109639) were purchase from GeneTex (USA). The signals collected from immunoblots were quantified by using Image Studio™ Lite Software.

### 2.7 Enzyme linked immunosorbent assay analysis

5.5 x 10^4^ C2C12 myoblasts were seeded per well in a 24-well plate. When reached 90% -100% confluence, the myoblasts were pre-treated with differentiation medium consisting a series concentration of GMI (0, 0.01, 0.05, 0.1, 0.5, 1, 5 and 10 μg/ml). The cultured medium were collected at different time points (24, 48 and 72 hours), and the levels of IL-6 were determined using ELISA kits (R&D Systems) according to the manufacturer’s instructions, and then quantified by Spark® Multimode Microplate Reader (Tecan, Switzerland) at O.D 450 nm.

### 2.8 Statistical analysis

The statistical analysis was performed by using GraphPad Prism 6 (GraphPad Software, California, USA). The datasets with multiple groups were analyzed by One-way ANOVA. The presented data are mean± SEM of three independent experiments (n = 3) the probability values less than 0.05 (p<0.05) were considered statistically significant.

## 3. Results

### 3.1 Induced myogenesis of C2C12 cells and primary myoblasts *in vitro*

**Fig 1:**
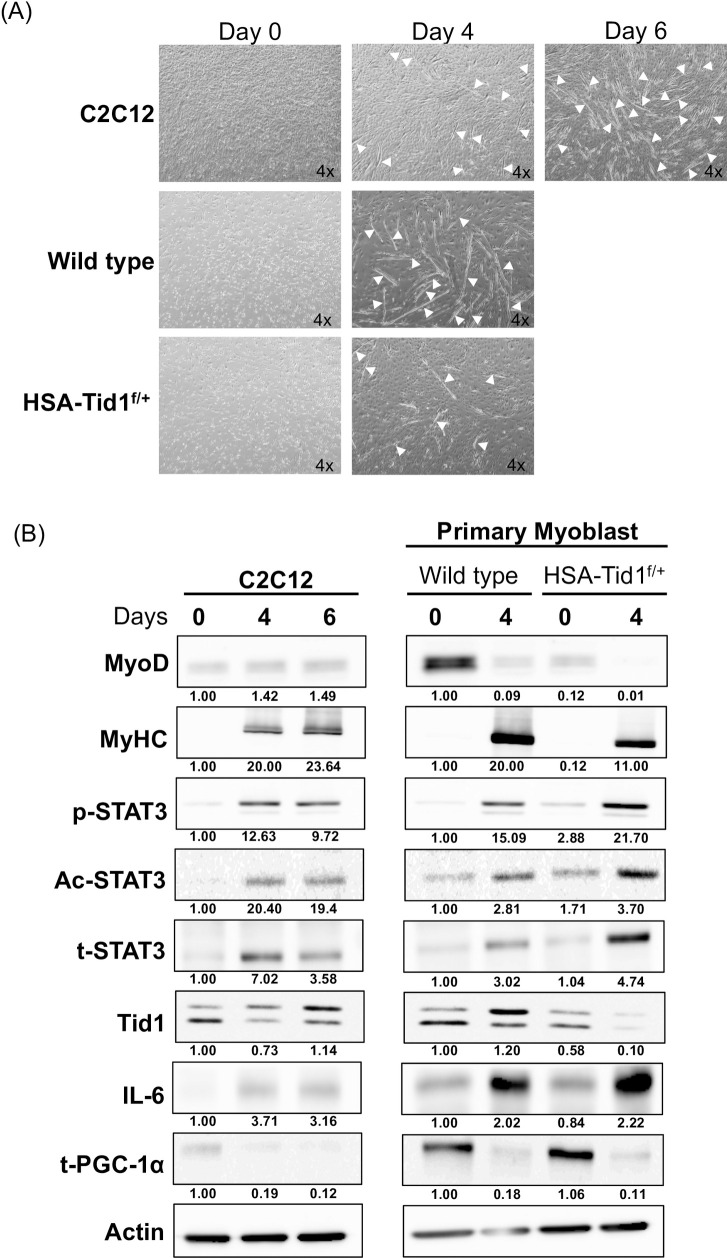
Differentiation of C2C12 and primary myoblast. (A) The representative phase contrast images of C2C12 cells and primary myoblasts under induced differentiation at different time intervals (Day 0, 4 and 6). White arrows indicating the myotube formation. (B) Immunoblot analyses showing the expression profile of myogenesis and mitochondrial biogenesis markers of C2C12 and primary myoblasts under induced differentiation (p-STAT3, phosphorylated STAT3; Ac, acetylated STAT3 and t-STAT3, total STAT3).

C2C12 cells and the isolated mice primary myoblast were grown under the differentiation medium and went on morphological change from mesenchymal cell type to extended stretched cell type as a myoblast along with cell fusion and elongation (Fig 1A). Immunoblotting analyses demonstrated that the expression profile of those known myogenesis markers, including MyoD, MyHC and STAT3 (both total- and phosphor-STAT3), which is similar to the previous findings [22] (Fig 1B). Strikingly, we observed the upregulation of acetylated-STAT3 in all the differentiated myoblasts (C2C12) and primary myoblasts (wild type and *HSA-Tid1*^*f/+*^), which has never been reported thus far (Fig 1B). Further, Tid1, a known mitochondrial protein acting as a tumor suppressor, was also up regulated in the differentiated C2C12 cells and the isolated primary myoblasts. However, the protein levels of Tid1 and MyHC in differentiated *HSA-Tid1*^*f/+*^ myoblasts were lower than that of wild-type differentiated myoblasts (Fig 1B). As shown in Fig 1A the number of isolated primary myoblasts from the *HSA-Tid1*^*f/+*^ mice were fewer than that isolated from the wild type mice. Of note, we observed that the expression level of MyoD and IL-6 of the C2C12 were less than that of wild type primary myoblasts (on both day 0 and day 4). In addition, we observed less total PGC-1α protein in the differentiated cell (C2C12 and primary myoblasts (wild type and *HSA-Tid1*^*f/+*^). This findings suggest that Tid1 protein is upregulated during myogenesis, and the Tid1 heterozygous deficiency leads to dysfunction of muscle tissue and these findings were consistent with our previous publication [22].

### 3.2 The cytotoxic effect of GMI on C2C12 myoblast

**Fig 2:**
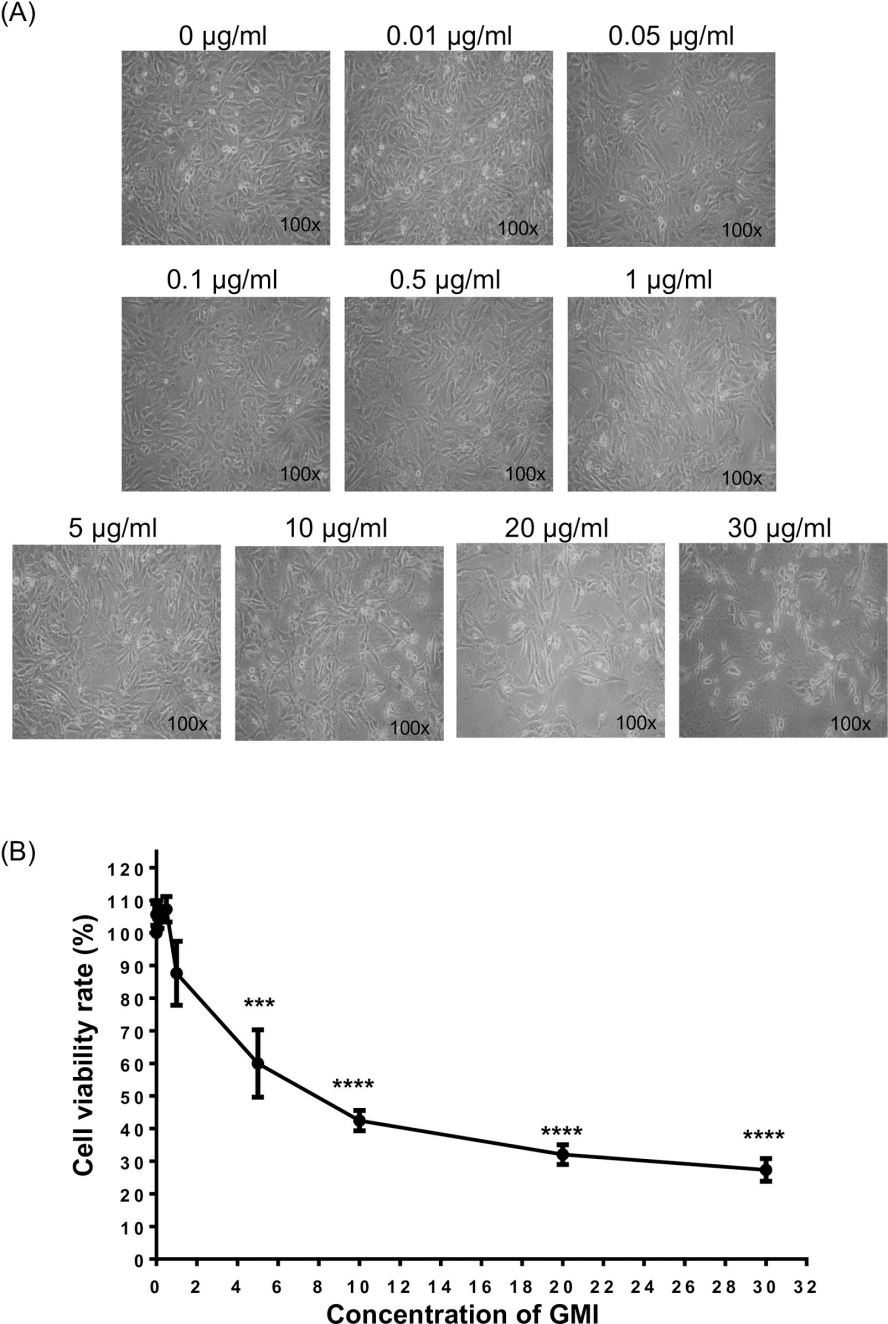
The cytotoxicity of GMI on C2C12 myoblasts. (A) C2C12 cells were treated with different dosage of GMI (0, 0.01, 0.05, 0.1, 0.5, 1, 5, 10, 20 and 30 μg/ml) for 24 hours and the morphological changes were visualized. (B) The cell viability of GMI pre-treated C2C12 was determined by using CCK-8 assay. Statistical analyses were performed by One-way ANOVA. ***, p < 0.001 and **** p < 0.0001.

Cytotoxicity is defined as the toxicity caused due to the action of drugs on living cells. Therefore, we tested the cytotoxic effect of GMI on the cell viability of C2C12 myoblasts. The concentration of GMI applied to the C2C12 myoblast were 0, 0.01, 0.05, 0.1, 0.5, 1, 5, 10, 20 and 30 μg/ml, respectively. After treatment with GMI for 24 hours, we visualized the cells morphology changes under microscopic (Fig 2A). We also observed that high concentration (> 10 μg/ml) of GMI affected the cell viability. Consequently, we determined the cell viability after treatment with GMI by using CCK-8 assay. The analytical results were consistent with the cells morphology changes, at which the high concentration of GMI (20 μg/ml and 30 μg/ml) caused a dramatic cell death (Fig 2B). The cell viability ratio of C2C12 myoblasts at 20 μg/ml and 30 μg/ml were 32 ± 5.42% and 27 ± 6.00% respectively. Contrary, GMI at lower concentration (0.01, 0.05, 0.1, 0.5 μg/ml) induced cell proliferation.

### 3.3 GMI pre-treatment promoting induced myogenesis in myoblast

**Fig 3:**
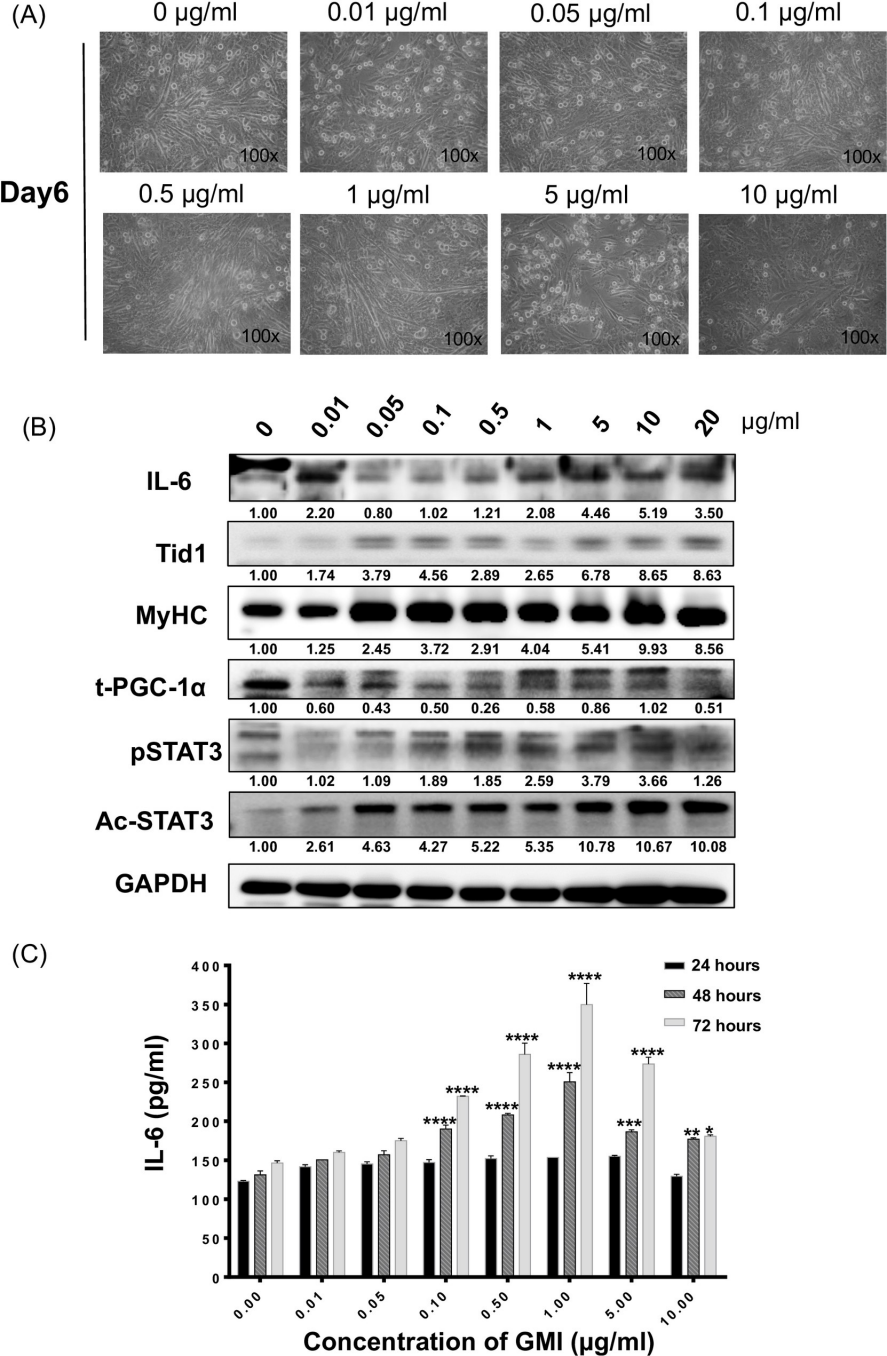
Promoted C2C12 myogenic differentiation with GMI pretreatment. (A) The representative phase contrast images of induced differentiated C2C12 with pretreatment of GMI (0, 0.01, 0.05, 0.1, 0.5, 1, 5,10 and μg/ml) on day 6. Immunoblot analyses showing the expression profile of myogenesis, mitochondrial biogenesis and pro-inflammatory markers of GMI treated C2C12 (B) crude cells lysate. (C) IL-6 secretion from C2C12 cells with GMI pre-treatment afterward 24, 48 and 72 hours was determined by ELISA assays. Data are means ± SEM. *P<0.05, **, p < 0.01 ***, p < 0.001 and **** p < 0.0001.

To assess the effects of GMI on promoting induced myogenesis in vitro, C2C12 cells of 90%-100% confluence were replaced with differentiation medium containing 0, 0.01, 0.05, 0.1, 0.5, 1, 5, 10, 20, 30 μg/ml of GMI for 24 hours. The GMI pre-treated C2C12 cells were set for further differentiation for 5 more days (Fig 3A). The morphology of C2C12 cells with GMI pre-treatment was recorded daily (data not shown). We observed that pre-treatment with GMI at high concentration, such as 20 and 30μg/ml, caused significant cell death (Fig 2). Nevertheless, pre-treatment of 0.05. 0.1, 0.5 and 1 μg/ml of GMI promoted the morphological change of C2C12 myoblast with cell fusion and elongation.

Crude cell extract proteins of GMI pre-treated C2C12 cells were collected on day 6. Immunoblotting analyses showed that GMI pretreatment with low concentration (0.01, 0.05, 0.1 and 0.5 μg/ml) inhibited the expression of IL-6 in differentiated C2C12 cells whereas pre-treatment with high dose of GMI (1, 5, 10 and 20 μg/ml) enhanced the expression of IL-6 (Fig 3B). We also observed that the expression profile of phosphorylated STAT3 and PGC-1α displayed a similar pattern as that of IL-6 (Fig 3B). Interestingly, pretreatment with GMI enhanced the expression of mitochondrial protein Tid1 and acetylated STAT3 (Ac-STAT3) in a dose-dependent manner (Fig 3B), especially at the lower concentration of GMI (0.01, 0.05, 0.1 and 0.5 μg/ml). Additionally, the specific myogenesis marker, MyHC, was also up regulated within the GMI pretreated C2C12 cells in a dose-dependent manner. Overall, the above results demonstrated that pretreatment of GMI can vary the activity of IL-6 and phosphor-STAT3 along with the up regulation of mitochondrial protein (Tid1) and biogenesis marker (MyHC).

We next determined the IL-6 secretion from GMI pre-treated C2C12 myoblasts using ELISA assays. The secretions of IL-6 were assessed at 24, 48 and 72 hours after GMI treatment (GMI (0, 0.01, 0.05, 0.1, 0.1, 0.5, 1, 5 and 10 μg/ml). The results revealed that there was not significant IL-6 secretion from cells treated with GMI for 24 hours. Nevertheless, we observed significant increment of IL-6 secretion of cells in a dose-dependent manner after 48- and 72 hours treatment of GMI, particularly at 0.1, 0.5 and 1 μg/ml (p < 0.0001) (Fig 3C). These data supported the notion that secretion of IL-6 was enhanced during C2C12 myoblasts differentiation after 48- and 72 hours at low dose of GMI treatment.

## 4. Discussion

Mammalian adult skeletal muscle is defined as stable tissue and possesses a remarkable ability to initiate a rapid and extensive repairing process to prevent the loss of muscle mass during injury [1–3]. Currently, many studies have proposed the use of human recombinant growth factors to induce the regeneration of skeletal muscle [39–41]. Insulin-like growth factor-1 (IGF-1) is found highly mitogenic for myoblasts [42–44]. Overexpression the human IGF-1 display muscle hypertrophy [45]. In addition, basic fibroblast growth factors (bFGF) and platelet-derived growth factors (PDGF), demonstrated a potent stimulating effects on satellite cell proliferation [40,41]. However, these growth factors may induce the production of transforming growth factor-beta (TGF-β1) which acts as deleterious agent for skeletal muscle myogenesis [46]. Recently, Shin and colleagues have shown that Red Ginseng extract induced the mitochondrial biogenesis and ATP production, consequently, promoting the differentiation of C2C12 myoblast [47]. Red Ginseng, a medicine herb, has been reported to possess the ability to protect muscle damage after strenuous exercise, relief fatigue and to upregulate the energy metabolism [48,49]. GMI, an immunomodulatory protein from *G*. *microsporum* is a traditional medicine and has been used for thousand years. Recently, most of the studies report the use of GMI in anti-tumor progression, inhibition of the proliferation of cancer cells and anti-inflammation. GMI inhibits epidermal growth factor (EGF)-induced metastasis through autophagy signaling and cause the cell death in lung cancer cells. GMI also been reported as a suppressor agent for oral carcinomas stem cells and, inhibit migration and invasion of lung cancer cell [26,27,29]. Concurrently, a study shows GMI could induce apoptosis in urinary bladder urothelial carcinoma cells [30]. Although the functions of GMI are well define in anti-cancerous, the study of GMI is rarely been countered on normal cells condition. In this study, we explored the promotion of GMI on myoblast myogenesis. We observed that pre-treatment with GMI successfully induced the C2C12 myoblast differentiation. We also observed the upregulating of specific myogenesis marker. Apart from that, we found upregulation of mitochondria protein Tid1, mitochondria biogenesis marker PGC-1α, and Ac-STAT3.

Regeneration of skeletal muscle is a highly synchronized process involving the activation of various cellular responses. Firstly, necrosis of damaged tissue and a cascade of inflammatory are activated, subsequently, followed by activation of myogenic cells to proliferate, differentiate and fuse to generate a new myofiber formation [2,3]. Many studies have reported that vary signaling factors are released during myogenesis of skeletal muscle. During skeletal muscle myogenesis, a continuous series of myogenic lineage is regulated by the positive or negative signals. Firstly, MyoD, the transcriptional activators of the myogenic regulatory factor family, is the upregulated [50–52]. Elevation of MyoD induces the proliferation of myoblast, subsequently follow by the downregulation of Pax7, a paired box transcription factor of satellite cells [4,53–55]. Kablar and colleagues have reported the total loss of skeletal muscle in MyoD ^-/-^ and Myf-5^-/-^ double knockout mice. Along with the myogenesis process, the proliferating myoblasts withdraw from the cell cycle and terminate the differentiation of myoblast. Consequently, the muscle-specific gene, myosin heavy chain (MyHC) is upregulated, where the terminally differentiated myoblasts fuse together and elongate to form a multinucleated muscle fiber [56–58]. Here, our data (Figs 1 and 3) showed the consistency with the previous publications.

In present study, we found that IL-6 signal was upregulated along with the myogenesis process (Fig 1B). Additionally, high dose GMI pre-treatment was able to increase the expression of intracellular IL-6 (Fig 3A) but low doses of GMI (0.1, 0.5 and 1 μg/ml) significantly induced the secretion of IL-6 (Fig 3B). Upregulation of IL-6 is essential for satellite cells proliferation [59]. Apart from that, IL-6 participates in myoblast differentiation and fusion. Hence, IL-6 is playing a dual role in myogenesis. For instance, the depletion of IL‐6 reduces the extent of myoblast differentiation and fusion. In opposite, genetic overexpression of exogenous IL-6 induced the myogenesis with elevation of expression of muscle specific genes expression. Myoblasts derived from IL‐6 null mice show inhibited differentiation and reduced fusion abilities. Although IL-6 is essentially needed in promoting differentiation, it also been reported that the activation of its downstream signaling molecule STAT3 is necessary to promote differentiation of myoblasts [11]. Wang et al. has reported the importance of JAK2/STAT2/STAT3 pathway in myogenic differentiation. They show that by individually knockdown the endogenous JAK2, STAT2, and STAT3, the differentiation of C2C12 myoblast is inhibited [60]. However, via the JAK1/STAT1/STAT3 pathway it could promote the myoblast differentiation [61]. In this study, we showed that the p-STAT3 is elevated when pre-treated with GMI. Nevertheless, upregulation of p-STAT3 was not dose-dependent. Surprisingly, we found the Ac-STAT3 signal was augmented with a dose-dependent manner along with GMI pre-treatment, particularly in low concentration of GMI (0.01, 0.05, 0.1 and 0.5 μg/ml), during C2C12 induced differentiation.

As abovementioned, the role of p-STAT3 in myogenesis is widely discussed. However, the role of STAT3 acetylation in myoblast myogenesis has never been determined. Acetylation of K685 STAT3 has been reported to facilitate the STAT3 dimerization and full transcriptional activity [62,63]. STAT3 acetylation is found to regulate the proliferation of cancer cell. CD44 is a type I transmembrane glycoprotein, through its C- terminal, to interact with N-terminal coiled-coil domain (NTD) of STAT3, then to alleviate the binding of p300 and to drive the STAT3 acetylation at K658. Subsequently, STAT3 acetylation activates Cyclin D1 promoter and induces the tumor proliferation [64]. STAT3 acetylation has been found to promote DNA methyltransferase 1 interactions (Dnmt1) expression in MEF cells [65]. In addition, DNMT1 is playing an important role in myogenic differentiation and cell fate transition [66]. A recent study shows that acetylated STAT3 is able to shuttle between cytosolic and mitochondria of lung cancer cells. Further, the constitutively acetylated STAT3 could translocate into mitochondrial and regulate the pyruvate metabolism for TCA cycle that helps to maintain the mitochondrial membrane potential and ATP synthesis [67].

Tid1 is expressed in two alternatively splicing isoforms, Tid1-L and Tid1-S which differ at the C- terminal tail. Interestingly, Lu *et al*. have highlighted the key differences between the subcellular localization dynamics of Tid1-L and Tid1-S. They also reveal that Tid1-L exhibit higher cytosolic stability and a slower rate of mitochondrial import compared with Tid1-S. They also reported that the interaction between Tid1 and STAT3 [14]. Furthermore, in our previous publication, we have reported the essential role of Tid1 in maintaining the integrity of mitochondrial and myoblast myogenic capacities. Tid1 deficiency can cause dysfunction of muscle tissue of transgenic mice *in vivo*. We also found that the 8-day old *HSA-Tid1*^*f/f*^ null mice showed Lordokyphosis phenotype and became postnatal lethality at postnatal day 8 to 10. Depletion of Tid1 suppresses C2C12 cell differentiation *in vitro*, which reduces the myotube formation. Moreover, Tid1 deletion impairs the mitochondria activity. The mitochondrial mass and membrane potential are abolished. Apparently, the mitochondrial biogenesis protein, PGC-1α is downregulated [22]. Above mentioned, isolated primary *HSA-Tid*^*f/+*^ myoblasts were poor differentiated and the myoblast numbers were fewer compared to the wild-type primary myoblasts (Fig 1A). Together, we postulated that upregulation of Tid1 along with its interacting protein, STAT3, undergoing acetylation during the myogenesis induced by GMI. In this study, we observed the expression of mitochondrial protein Tid1 were upregulated in GMI pre-treatment differentiated myoblast. Hence, Tid1 plays a pivotal role in myogenic process and muscle regeneration.

PGC-1α is required for the induction of many antioxidant-detoxifying enzymes and acts as a modulator to coordinate the skeletal muscle for adaption of exercise [68,69]. Skeletal muscle specific overexpression PGC-1α mice demonstrates enrichment of type I myofibers, a fast glycolytic muscle and facilitates the switch of oxidative metabolism, consequently, that promotes the muscle performance and reduces the muscle fatigue [35]. PGC-1α also has been reported to regulate skeletal muscle mass, particularly in condition of muscle atrophy [70]. Nevertheless, we did not observed the upregulation of PGC-1α protein level during C2C12 differentiation. Hence, we speculated that the limited detection of PGC-1α protein may be caused from unknown post-translational modification.

Sarcopenia, an age-related skeletal muscle loss and dysfunction is public health issue since the average life span is increasing these years. As ageing progresses, mitochondria dysfunction in skeletal muscle reduced the capabilities of muscle regeneration [71]. Thus ageing results the impairment of muscle contractile and muscular atrophy [72,73]. Moreover, during ageing, the abnormalities of metabolic or endocrines bring to chronic inflammation. The pro-inflammatory mediator such as TNF-α and nuclear factor-κB (NF-κB) induce the apoptotic cell death and reduce the myogenesis capabilities [74,75]. Above mentioned, GMI possess the anti-inflammatory effect, thus might suppress the ageing-related inflammation response. In additional, we had demonstrated that application of low concentration of GMI could induce myoblast differentiation and along with the upregulation of the mitochondrial protein Tid1. Collectively, our findings suggest that GMI may have the utility of skeletal muscle regeneration.

**Fig 4:**
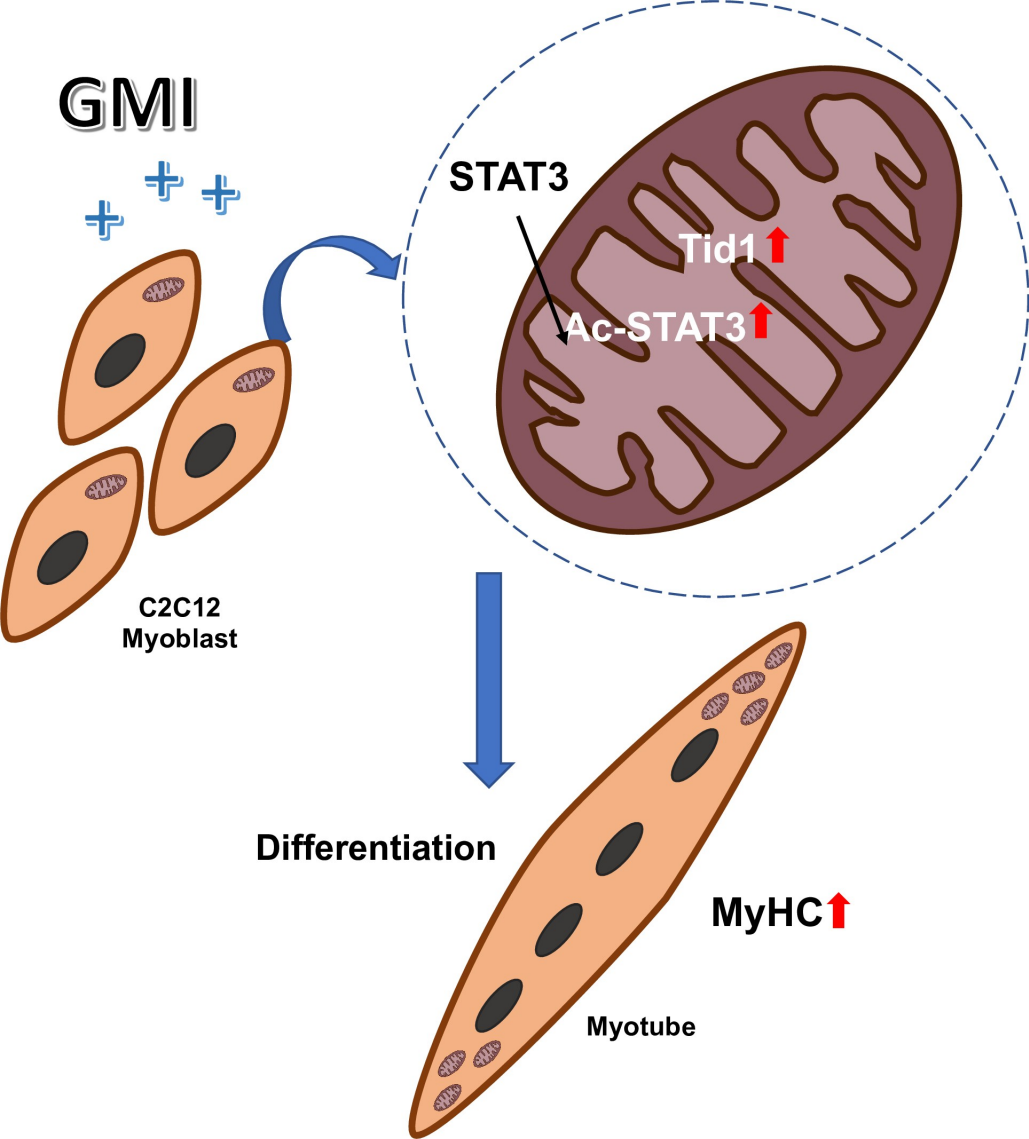
Schematic depicts that GMI pretreatment promotes C2C12 myogenic differentiation via activation of Tid1 and Ac-STAT3.

In summary, our results demonstrate that Tid1 might play a pivotal role in regulating GMI induced myoblast differentiation. We propose that pre-treatment of GMI promotes the myogenic differentiation via upregulation of Tid1 and Ac-STAT3 (Fig 4).

## Supporting information

S1 File (RAR)

S2 File (RAR)
